# Scion–Rootstock Interactions Enhance Freezing Stress Resilience in *Citrus reticulata* Through Integrated Antioxidant Defense and Carbon–Nitrogen Metabolic Adjustments

**DOI:** 10.3390/plants14193029

**Published:** 2025-09-30

**Authors:** Alaiha Asif, Shahid Iqbal, Carlos Eduardo Aucique-Perez, KeAndre Leaks, Rashad Mukhtar Balal, Matthew Mattia, John M. Chater, Muhammad Adnan Shahid

**Affiliations:** 1 Horticultural Science Department, North Florida Research and Education Center, IFAS/University of Florida, Quincy, FL 32351, USA; alaihaasif@ufl.edu (A.A.); shahidiqbal@ufl.edu (S.I.); c.auciqueperez@ufl.edu (C.E.A.-P.); leaks.k@ufl.edu (K.L.); 2 Department of Horticulture, College of Agriculture, University of Sargodha, Sargodha 40100, Pakistan; uaf_rashad@yahoo.com; 3 United States Horticultural Research Laboratory, United States Department of Agriculture, Agricultural Research Service, 2001 South Rock Road, Fort Pierce, FL 34945, USA; matthew.mattia@usda.gov; 4 Horticultural Science Department, Citrus Research and Education Center, IFAS/University of Florida, Lake Alfred, FL 33850, USA; jchater@ufl.edu

**Keywords:** antioxidants, freeze tolerance, photosynthesis, rootstock, reactive oxygen species

## Abstract

Frequent and increasingly severe freezing events threaten citrus production in northern Florida, underscoring the need for strategies that enhance freezing resilience in citrus cultivars. Grafting scions onto tolerant rootstocks provides a physiologically integrative approach to improve stress tolerance. This study aims to elucidate how these interactions modulate physiological and metabolic responses under freezing stress, thereby identifying mechanisms that contribute to enhanced freeze resilience in citrus. Here, we grafted *Citrus reticulata* (cv. UF-950) onto eight rootstocks (Bitters, Blue-1, C-146, Sour Orange, UFR07TC, UFR09TC, UFR5, and US942) to evaluate scion–rootstock interactions under normal (20 °C) and freezing (−6 °C) conditions. Freezing stress caused a sharp increase in oxidative stress markers, lipid peroxidation, and membrane damage while reducing photosynthetic performance across most combinations. Antioxidant capacity, osmolyte accumulation, and carbon–nitrogen metabolic responses varied significantly among rootstocks, revealing strong genotype-dependent modulation of scion physiology. Among the tested combinations, UF-950 grafted onto UFR5 displayed the highest freezing tolerance, characterized by robust activation of antioxidant enzymes, elevated proline and glycine betaine accumulation, reduced oxidative damage, and sustained carbon–nitrogen metabolic fluxes under freezing stress. These results demonstrate that rootstock genotype governs the extent of scion defense activation and metabolic homeostasis under freezing conditions. Our findings identify UFR5 as a promising rootstock for enhancing freezing resilience in citrus and provide mechanistic insight into how scion–rootstock interaction orchestrates integrative stress tolerance pathways. Future work should focus on multi-omics dissection of rootstock-mediated signaling networks and long-term field validation to optimize rootstock selection for enhanced cold resilience under variable climatic conditions.

## 1. Introduction

Climate change and global warming are amplifying the intensity and frequency of abiotic stresses by driving more extreme and unpredictable weather patterns, including prolonged droughts, heavy rainfall, rising global temperatures, and increased occurrences of both cold and heat waves [1]. Among these, cold and freezing stress poses a significant threat to plant growth and survival, particularly for tropical and subtropical species that are inherently less adapted to low temperatures [2]. The impact of cold stress is especially critical as it limits not only plant productivity but also determines the geographical distribution of many species. In agronomic systems, cold stress is typically classified into three temperature regimes: freezing stress (below 0 °C), chilling stress (from 0 °C up to the minimum growth threshold), and suboptimal temperatures (between the growth threshold and the crop’s optimum temperature), each with distinct physiological implications for plant function [3]. Under stress conditions, plants are subjected to morphophysiological, biochemical, metabolic, and molecular disruptions, resulting in reductions in plant growth and yield [4,5]. Freezing stress affects the primary metabolism in plants due to a significant reduction in the CO_2_ assimilation, evidencing a lower rate of photosynthesis, transpiration, and stomatal closure, as well as the decrease in enzymatic activity of the Calvin cycle coupled to alterations in carbohydrate and amino acid levels [6,7,8,9]. Under physiological and metabolic changes, both photoinhibition and photooxidative damage were induced, impacting the structure and functionality of plant cell organelles, such as the chloroplast and mitochondria [9,10].

One of the most detrimental effects of freezing stress is oxidative damage, which is caused by an excess of reactive oxygen species (ROS) because of the disruption of the photosynthetic machinery [11,12]. Studies on citrus plants under cold stress have reported cellular instability resulting from increased levels of H_2_O_2_ and O_2_^•−^ [12,13,14]. Antioxidants such as superoxide dismutase (SOD), catalase (CAT), and ascorbate peroxidase (APX) play a crucial role in mitigating reactive oxygen species (ROS) accumulation during freezing stress, thereby protecting cellular membranes and maintaining redox balance [15,16]. In parallel, osmolytes including proline, glycine betaine, and soluble sugars (e.g., sucrose and raffinose) contribute to osmotic adjustment, stabilization of proteins and membranes, and prevention of ice crystal formation [17,18,19]. To mitigate the detrimental effects of freezing stress, the strategic deployment of scion–rootstock combinations has emerged as a practical approach in fruit crop improvement programs. This technique enhances the adaptability and resilience of horticultural species under adverse environmental conditions. Numerous studies have demonstrated that specific scion–rootstock interactions can significantly influence plant physiological responses, thereby improving tolerance to various abiotic stresses [20,21,22,23].

In citrus, Balfagón et al. [24] reported that the Carrizo citrange (*Citrus sinensis × Poncirus trifoliata*) rootstock exhibited superior tolerance to combined drought and heat stress compared to Cleopatra mandarin (*C. reshni*), primarily due to a stronger induction of the antioxidant defense system, which alleviated oxidative damage. Similarly, Bañuls and Primo-Millo [25] evaluated Clementine (*C. clementina Hort. ex Tan.*) and Navel orange [*C. sinensis* (L.) Osbeck] scions grafted onto Cleopatra mandarin and Troyer citrange rootstocks under water-deficit conditions. Their findings revealed that scions grafted onto Cleopatra mandarin accumulated significantly lower levels of chloride ions (Cl^−^) in leaves than those on Troyer citrange, indicating a rootstock-regulated ion exclusion mechanism. Enhanced cold tolerance has also been observed in crops such as apple, tomato, and cucumber using compatible scion–rootstock pairings [26,27]. Beyond stress tolerance, rootstocks also play a critical role in enhancing water and nutrient uptake efficiency [28,29]. However, the selection of an optimal rootstock must be tailored to the specific agro-climatic conditions of the cultivation region [30]. Accordingly, diversifying scion–rootstock combinations remains a central objective of global rootstock breeding initiatives, which aim at improving fruit quality, increasing yield stability, and extending the harvest period [31,32,33,34].

*Citrus reticulata,* commonly known as mandarin, tangerine, Unshu orange, or Comola in the Asian subcontinent, is a widely cultivated citrus species valued for its sweet, juicy, and nutritious fruit [35,36]. Grown in tropical and subtropical regions worldwide, its production has recently declined due to unexpected freezing events occurring during winter and early spring. With increasing climate variability, the frequency and severity of such events, the development of cold-resilient grafting combinations is essential for sustaining citrus cultivation in low-temperature regions [12].

Therefore, the objective of this study is to provide a comprehensive evaluation of the physiological and metabolic responses of different scion–rootstock combinations under freezing stress. Although rootstock choice is known to influence citrus growth, vigor, and stress adaptation, the specific mechanistic contribution of scion–rootstock interactions to freezing stress tolerance has remained unexplored. Previous studies have primarily focused on individual stress responses, such as antioxidant activity, osmolyte accumulation, or carbohydrate metabolism, without integrating these pathways across grafted combinations. Moreover, the role of carbon–nitrogen metabolic reprogramming as a coordinated response to freezing stress in citrus scion–rootstock systems has not been clearly defined. Therefore, this study fills that gap by demonstrating how UF950 mandarin scion grafted onto different rootstock enhances freezing resilience through the integration of antioxidant defense, osmotic balance, and carbon–nitrogen metabolism, providing novel insight into the physiological and biochemical basis of rootstock-mediated freezing tolerance. The insights gained from this study will contribute to a deeper understanding of the mechanisms underlying cold hardiness in citrus and offer valuable guidance for breeding programs targeting the development of cold-tolerant cultivars.

## 2. Results

### 2.1. Analysis of Variance

The effects of rootstocks, freezing stress, and their interactions were statistically significant for multiple physiological and metabolic traits (Supplementary Table S1). Freezing stress markedly reduced photosynthetic performance, with substantial declines in assimilation rate (*A*), stomatal conductance (*g*_s_), and leaf greenness (SPAD). Stress exposure also triggered a sharp increase in ROS accumulation (O_2_^•–^ and H_2_O_2_), which coincided with elevated lipid peroxidation (LPO) and electrolyte leakage (EL), indicating enhanced membrane damage. Antioxidant enzyme activities (superoxide dismutase—SOD, peroxidase—POD, catalase—CAT, ascorbate peroxidase—APX, glutathione peroxidase—GPX, glutathione reductase—GR, monodehydroascorbate reductase—MDAR, dehydroascorbate reductase—DHAR) and non-enzymatic antioxidants (glutathione—GSH) were significantly upregulated. However, the magnitude of response varied among rootstocks. Similarly, osmolytes (proline and glycine betaine) accumulated to higher levels under freezing conditions, contributing to osmoprotection. Carbon metabolism was significantly altered, with reductions in glucose, fructose, sucrose, starch, and TSS concentrations, accompanied by shifts in carbohydrate-metabolizing enzyme activities (acid invertase-AI, neutral invertase-NI, sucrose synthase-SuSy, sucrose phosphate synthase-SPS, fructokinase-FK, phosphofructokinase-PFK, hexokinase-HK, and pyruvate kinase-PK activities). Nitrogen metabolism was also affected, with freezing stress significantly suppressing NR and NIR activities. Notably, rootstock choice influenced both the intensity of stress-induced damage and the capacity for antioxidant, osmolyte, and C-N metabolic adjustments.

### 2.2. Photosynthetic Capacity and Leaf Greenness

In the present study, no significant differences were observed among assimilation rate (*A*), stomatal conductance (*g*_s_), and leaf greenness (SPAD) in plants grafted onto eight different rootstocks under normal conditions (20 °C), but when these plants were subjected to FZS, a significant difference was observed. Citrus rootstocks subjected to FZS showed substantial reductions of 20 to 60% for *A* and *g*_s_ and of 5 to 40% for the SPAD index when compared to citrus rootstocks under normal conditions (Table 1). For *A* under FZS, the only significant difference was observed in UF950 plants grafted onto rootstock UFR5 (18.5 µmol photons m^−2^ s^−1^) and sour orange (8.3 µmol photons m^−2^ s^−1^). The *g*_s_ and SPAD values were also significantly higher in UF950 plants grafted on UFR5 (44.8 mol CO_2_ m^−2^ s^−1^ and 48.2, respectively) as compared to UF950 plants grafted on sour orange (20.5 mol CO_2_ m^−2^ s^−1^ and 29.4, respectively) under freezing stress. Overall, these results highlight the crucial role of rootstock selection in modulating photosynthetic performance and leaf function under freezing stress.

### 2.3. Freezing Stress Influences Oxidative Stress and Membrane Damage

**Figure 1:**
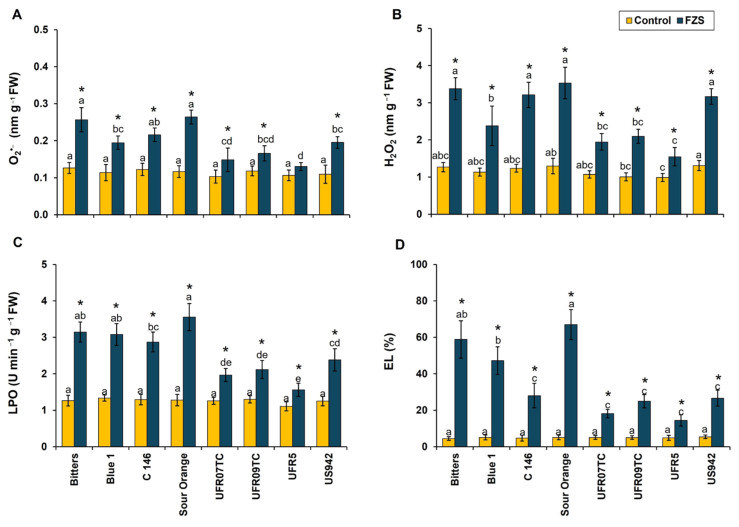
Response of oxidative stress [superoxide ion (O_2_^•−^; (**A**)), hydrogen peroxide (H_2_O_2_; (**B**)), lipid peroxidation (LPO; (**C**)), and electrolyte leakage (EL; (**D**))] on leaves of eight citrus rootstocks submitted to two temperature levels [20 °C (control) and −6 °C (freezing stress)]. Mean values (*n* = 4) marked with an asterisk (*) indicate significant differences between temperature levels as determined by the F-test (*p* ≤ 0.05). For each temperature level, means (*n* = 4) followed by different letters denote significant differences among rootstocks according to Tukey’s test (*p* ≤ 0.05), with bars representing the standard deviation; FW = fresh weight.

In the present study, superoxide anion (O_2_^•−^) production was significantly elevated in all genotypes under FZS compared with their respective controls (Figure 1A). The highest O_2_^•−^ accumulation was observed in sour orange, bitters, C-146 and Blue-1 (0.26, 0.25, 0.21 and 0.19 nm g^−1^ FW, respectively), where levels reached nearly threefold those of the control (0.11, 0.12, 0.12 and 0.11, nm g^−1^ FW, respectively). By contrast, UFR5 and UFR07TC exhibited comparatively lower increases in O_2_^•−^ (0.13 and 0.14 nm g^−1^ FW, respectively), suggesting a reduced ROS burst in these genotypes. Similarly, hydrogen peroxide (H_2_O_2_) content followed a similar trend, with FZS markedly enhancing H_2_O_2_ levels across all genotypes (Figure 1B). Among them, sour orange, bitters, and C-146 accumulated the highest H_2_O_2_ concentrations (3.53, 3.38 and 3.21 nmol g^−1^ FW, respectively), whereas UFR5 and UFR07TC maintained relatively lower levels (1.54 and 1.94 nmol g^−1^ FW, respectively), indicating a potential genotype-dependent ROS detoxification capacity.

Consistent with elevated ROS, lipid peroxidation (LPO), measured as malondialdehyde equivalents, increased significantly under FZS (Figure 1C). The most severe peroxidative damage occurred in sour orange and bitters (3.55 and 3.14 U min^−1^ g^−1^ FW, respectively), while UFR5 and UFR07TC maintained significantly lower LPO levels (1.56 and 1.96 U min^−1^ g^−1^ FW, respectively), reflecting enhanced membrane stability under freezing stress. For electrolyte leakage (EL), a direct indicator of plasma membrane integrity, was dramatically higher in FZS-treated plants compared with controls (Figure 1D). Sour Orange exhibited the greatest EL (66.96%), followed by Bitters (58.82%) and Blue-1 (47.20%). In contrast, the lowest EL was observed in UFR5 and UFR09TC (14.4 and 18.14%, respectively). Notably, the superior membrane stability in these genotypes corresponded with their reduced ROS accumulation and LPO levels, highlighting their enhanced tolerance to freezing-induced oxidative stress. These findings demonstrate pronounced genotype-dependent variation in ROS accumulation and membrane stability under freezing stress. The comparatively lower ROS generation, reduced lipid peroxidation, and limited electrolyte leakage in UFR5 and UFR07TC indicate that these rootstock–scion combinations possess superior oxidative stress tolerance mechanisms relative to the highly susceptible genotypes such as Sour Orange and Bitters.

### 2.4. Contribution of Differential Antioxidant Capacity to Variation in Freezing Stress Among Scion–Rootstock Combinations

**Figure 2:**
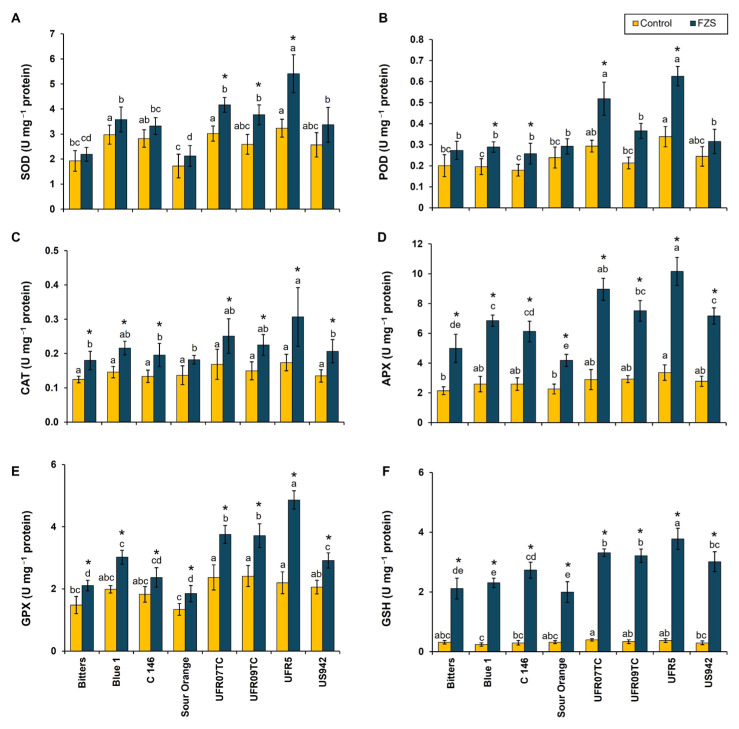
Response of antioxidant enzyme activities [superoxide dismutase (SOD; (**A**)), peroxidase (POD; (**B**)), catalase (CAT; (**C**)), ascorbate peroxidase (APX; (**D**)), glutathione peroxidase (GPX; (**E**)), and glutathione (GSH; (**F**))] on leaves of eight citrus rootstocks submitted to two temperature levels [20 °C (control) and −6 °C (freezing stress)]. Mean values (*n* = 4) marked with an asterisk (*) indicate significant differences between temperature levels as determined by the F-test (*p* ≤ 0.05). For each temperature level, means (*n* = 4) followed by different letters denote significant differences among rootstocks according to Tukey’s test (*p* ≤ 0.05), with bars representing the standard deviation; U = units.

In the present study, superoxide dismutase (SOD) activity increased significantly in all genotypes following FZS, with the most potent induction observed in UFR5 and UFR07TC (5.41 and 4.16 U mg^−1^ protein, respectively). In contrast, susceptible genotypes such as Bitters, C-146, and Blue-1 showed comparatively modest increases (2.18, 3.32, and 3.58 U mg^−1^ protein, respectively), while Sour Orange maintained intermediate levels (2.12 U mg^−1^ protein). These results indicate that enhanced SOD activity contributes to more efficient dismutation of O_2_^−^ radicals in tolerant genotypes (Figure 2A). Peroxidase (POD) activity was also markedly elevated under FZS across all rootstock–scion combinations. The highest POD activity was recorded in UFR5 and UFR07TC (0.62 and 0.51 U mg^−1^ protein, respectively), whereas Blue-1 and C-146 exhibited the lowest induction (0.28 and 0.25 U mg^−1^ protein, respectively). This suggests that the tolerant genotypes maintain superior peroxidative capacity to detoxify H_2_O_2_ generated during freezing stress (Figure 2B).

Catalase (CAT) activity showed a pronounced genotype-dependent response to FZS. While CAT activity doubled in UFR5 and UFR07TC (0.30 and 0.25 U mg^−1^ protein, respectively), only marginal increases were detected in C-146 and Sour Orange (0.19 and 0.18 U mg^−1^ protein, respectively (Figure 2C). The higher CAT activity in tolerant genotypes reflects an enhanced ability to scavenge H_2_O_2_ through dismutation into water and oxygen directly. Similarly, ascorbate peroxidase (APX) activity was significantly higher in all genotypes, with the highest induction observed in UFR5 (10.15 U mg^−1^ protein), followed by UFR07TC (8.95 U mg^−1^ protein). In contrast, C-146 and Bitters maintained low APX activities (6.12 and 4.98 U mg^−1^ protein, respectively), with the lowest in Sour Orange (4.17 U mg^−1^ protein). Given the significant role of APX in the ascorbate-glutathione cycle, this enhanced activity indicates improved fine-tuned H_2_O_2_ detoxification in tolerant combinations (Figure 2D).

Glutathione peroxidase (GPX) activity exhibited a similar trend, with tolerant genotypes UFR5 and UFR07TC showing the highest activity (4.86 and 3.75 U mg^−1^ protein, respectively). Conversely, Sour Orange and Bitters displayed the lowest GPX activity (1.85 and 2.10 U mg^−1^ protein, respectively) under freezing stress, indicating limited glutathione-dependent ROS scavenging (Figure 2E). The reduced glutathione (GSH) content increased significantly in all genotypes under FZS, with the most pronounced accumulation in UFR5 and UFR07TC (3.77 and 3.31 U mg^−1^ protein, respectively). By comparison, Sour Orange and Bitters accumulated less GSH (1.99 and 2.11 U mg^−1^ protein, respectively), suggesting a weaker redox buffering capacity (Figure 2F). Overall, the consistently higher activities of SOD, CAT, APX, GPX, and greater GSH accumulation in UFR5 and UFR09TC highlight their superior antioxidative defense machinery, which underpins their enhanced tolerance to freezing-induced oxidative stress.

### 2.5. Accumulation of Osmolytes Across Scion–Rootstock Combinations Under Freezing Stress

**Figure 3:**
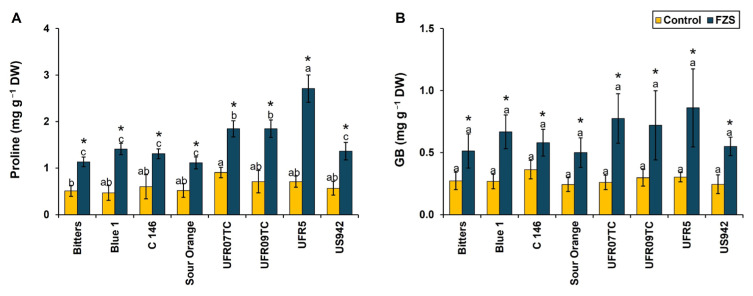
Response of osmolytes accumulation [Proline (**A**), and glycine betaine GB; (**B**)] on leaves of eight citrus rootstocks submitted to two temperature levels [20 °C (control) and −6 °C (freezing stress)]. Mean values (*n* = 4) marked with an asterisk (*) indicate significant differences between temperature levels as determined by the F-test (*p* ≤ 0.05). For each temperature level, means (*n* = 4) followed by different letters denote significant differences among rootstocks according to Tukey’s test (*p* ≤ 0.05), with bars representing the standard deviation; DW = dry weight.

In this study, we observed that FZS significantly influenced the accumulation of osmoprotectants, including proline and glycine betaine (GB) (Figure 3). In the case of proline accumulation, all rootstock combinations showed a significant increase under FZS compared with the control. The extent of accumulation, however, varied across genotypes. The most pronounced increase was observed in scions grafted onto UFR5, which exhibited a threefold rise (2.71 mg g^−1^ DW) relative to the control 0.71 mg g^−1^ DW), making it the highest proline-accumulating combination. This was followed by UFR07TC and UFR09TC, both of which also displayed substantial increases (1.84 and 1.83 mg g^−1^ DW, respectively). Intermediate proline levels were recorded in plants grafted on Blue-1 (1.41 mg g^−1^ DW), C-146 (1.30 mg g^−1^ DW), and US-942 (1.36 mg g^−1^ DW), whereas the lowest, yet still significant, increase was observed in Sour orange and Bitters rootstocks (1.11 and 1.13 mg g^−1^ DW, respectively) (Figure 3A). These findings highlight apparent genotypic variation in rootstock-mediated osmotic adjustment through proline biosynthesis during freezing stress.

Similarly, GB accumulation was consistently elevated under FZS across all rootstocks compared with their respective controls (Figure 3B). The magnitude of increase was again highest in the UFR5 combination (0.86 mg g^−1^ DW) under stress. UFR07TC and UFR09TC (0.77 and 0.72 mg g^−1^ DW, respectively) also exhibited marked increases, significantly higher than control levels (0.26 and 0.29 mg g^−1^ DW, respectively). In contrast, Sour orange and Bitters rootstocks showed more modest increases (0.5 and 0.51 mg g^−1^ DW, respectively), though the stress effect was still statistically significant. Notably, the relative ranking of rootstocks for GB accumulation closely paralleled that observed for proline, indicating a coordinated osmoprotectants response modulated by rootstock genotype. Overall, these results demonstrate that rootstock genotype exerts a strong influence on the capacity of scions to accumulate osmoprotectants under freezing stress. Notably, UFR5 consistently supported the highest accumulation of both proline and GB, suggesting that this rootstock confers superior osmotic adjustment and stress resilience to the scion. The substantial increases in osmolyte accumulation across genotypes further support the notion that enhanced osmoprotectant metabolism is a principal component of freezing tolerance in citrus.

### 2.6. Nitrogen Metabolism Changes in Citrus Rootstock Under Freezing Stress

**Figure 4:**
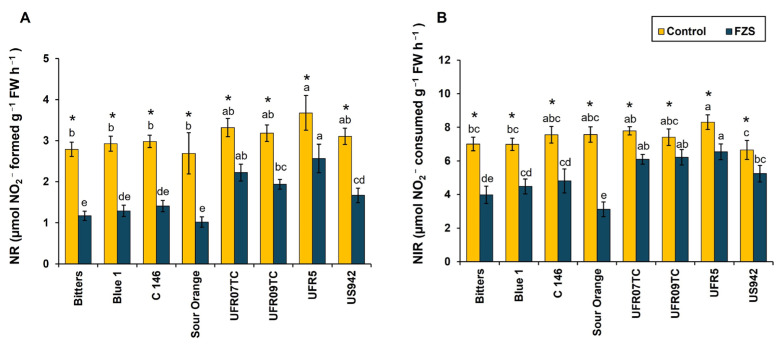
Response of the activities of enzymes involved in the nitrogen [nitrate reductase (NR; (**A**)) and nitrite reductase (NIR; (**B**))] on leaves of eight citrus rootstocks submitted to two temperature levels [20 °C (control) and −6 °C (freeze stress)]. Average values with an asterisk (*) differ significantly (*p* ≤ 0.05) between temperature levels according to the *F* test. Meanwhile, rootstock average values for each temperature level followed by different letters are significantly different according to Tukey’s test (*p* ≤ 0.05). Bars represent the standard deviation; *n* = 4; DW = dry weight.

In the present study, the concentration of NR and NIR is higher in all rootstocks combinations under control conditions as compared to FZS (Figure 4). Under normal conditions, a significant difference is observed in value of NR between UF950 plants grafted onto UFR5 (3.67 µmol NO_2_^−^ formed g^−1^ FW h^−1^) with Sour orange (2.69 µmol NO_2_^−^ formed g^−1^ FW h^−1^), C-146 (2.98 µmol NO_2_^−^ formed g^−1^ FW h^−1^), Blue-1 (2.92 µmol NO_2_^−^ formed g^−1^ FW h^−1^) and Bitters (2.78 µmol NO_2_^−^ formed g^−1^ FW h^−1^). A decrease in the concentration of NR is observed under FZS as compared to the control condition. The significantly lower concentration was observed in Sour orange (1.01 µmol NO_2_^−^ formed g^−1^ FW h^−1^), Bitters (1.17 µmol NO_2_^−^ formed g^−1^ FW h^−1^), Blue-1 (1.29 µmol NO_2_^−^ formed g^−1^ FW h^−1^) and C-146 (1.40 µmol NO_2_^−^ formed g^−1^ FW h^−1^) as compared to UFR5 (2.56 µmol NO_2_^−^ formed g^−1^ FW h^−1^), which has a significantly higher concentration of NR under FZS (Figure 4A).

In case of NIR, normal conditions showed significantly higher concentrations in UFR5 (8.30 µmol NO_2_^−^ consumed g^−1^ FW h^−1^) as compared to US942 (6.65 µmol NO_2_^−^ consumed g^−1^ FW h^−1^), Blue-1(6.98 µmol NO_2_^−^ consumed g^−1^ FW h^−1^), and Bitters (7.00 µmol NO_2_^−^ consumed g^−1^ FW h^−1^). In comparison, these concentrations decreased when plants were subjected to FZS. The significantly lowest concentration of NIR is observed in Sour orange (3.12 µmol NO_2_^−^ consumed g^−1^ FW h^−1^) and Bitters (3.98 µmol NO_2_^−^ consumed g^−1^ FW h^−1^) under FZS (Figure 4B). So, freezing stress profoundly affects NI and NIR by suppressing reductase activity and improving proline synthesis, which helps to enhance osmotic adjustments and antioxidant defense, thereby improving freezing tolerance.

### 2.7. Differential Regulation of Carbon Metabolism Enzymes Across Scion–Rootstock Combinations Under Freezing Stress

**Figure 5:**
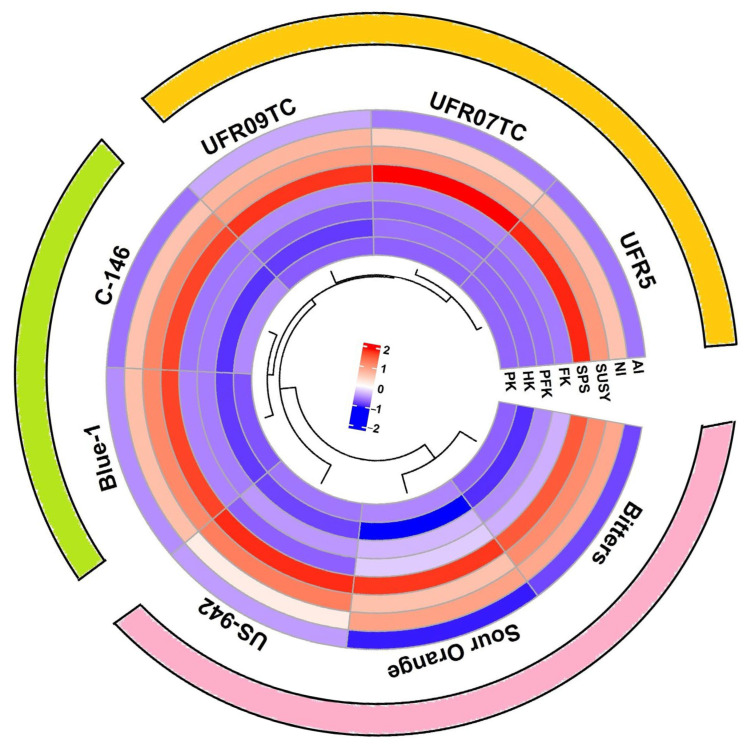
A clustered circular heatmap illustrate the activities of sucrose metabolism enzymes [acid invertase (AI); neutral invertase (NI); sucrose synthase (SUSY) and sucrose phosphate synthase (SPS)] and glycolytic enzymes [hexokinase (HK); fructokinase (FK); phosphofructokinase (PFK) and pyruvatekinase (PK) on leaves of eight citrus rootstocks submitted to two temperature levels [20 °C (control) and −6 °C (freeze stress)]. Color gradients represent normalized enzyme activities (log2-transformed), with red indicating upregulation and Blue-1 indicating downregulation relative to controls. Hierarchical clustering grouped as freeze-tolerant rootstocks in the yellow outer ring (UFR5, UFR07TC, UFR09TC), moderately tolerant rootstocks in the green outer ring (Blue-1-1, C-146), and freeze-sensitive rootstocks in the pink outer ring (Sour Orange, Bitters, US-942) clustered separately due to their suppressed metabolic responses.

Freezing stress markedly altered the activities of key enzymes involved in carbohydrate-related metabolic pathways across citrus rootstock, with distinct differences among genotypes shown as a clustered circular heatmap (Figure 5 and Supplementary Table S2). Clear genotype-specific patterns emerged, highlighting the significant role of rootstock background in shaping metabolic responses to freezing stress. Combinations involving UFR5, UFR07TC, and UFR09TC consistently exhibited strong upregulation of sucrose-metabolizing enzymes, including neutral invertase (NI), sucrose synthase (SuSy), and sucrose phosphate synthase (SPS). Enhanced sucrose cleavage was accompanied by elevated activities of glycolytic enzymes such as fructokinase (FK), phosphofructokinase (PFK), hexokinase (HK), and pyruvate kinase (PK), suggesting efficient channeling of soluble sugars into energy metabolism to sustain stress responses.

By contrast, Bitters, Sour Orange, and US-942 rootstocks displayed markedly reduced activity across most carbon metabolic enzymes, with pronounced downregulation of SuSy, SPS, and glycolytic steps, indicative of metabolic suppression under freezing stress. Intermediate patterns were observed in Blue-1 and C-146, where moderate increases in AI and SPS activity were detected, but glycolytic flux remained comparatively lower than in UFR-series rootstocks. Hierarchical clustering further grouped the tolerant UFR-series rootstocks (Figure 5), reflecting their coordinated metabolic reprogramming, while sensitive rootstocks clustered separately due to their limited or negative enzyme regulation. Overall, the freezing tolerance is strongly associated with the capacity to maintain sucrose turnover and glycolytic energy supply, processes most prominently activated in UFR5 and UFR07TC combinations.

### 2.8. Rootstock Type-Mediated Sugar Accumulation in Citrus Rootstocks Under Freezing Stress

Freezing stress (FZS) strongly altered the profiles of soluble sugars and starch in citrus rootstocks compared with normal condition plants (Table 2). Glucose concentrations increased significantly under FZS in all rootstocks. The most significant increases were observed in UFR5 (from 16.71 to 54.98 mg g^−1^ DW), followed by UFR07TC (17.05 to 46.37 mg g^−1^ DW) and UFR09TC (14.96 to 42.60 mg g^−1^ DW), whereas Sour Orange showed the lowest glucose accumulation (20.29 mg g^−1^ DW). Fructose levels were also strongly enhanced by FZS, with UFR5 again showing the most significant accumulation (23.31 mg g^−1^ DW), followed by UFR07TC (19.54 mg g^−1^ DW) and UFR09TC (17.78 mg g^−1^ DW). In contrast, Bitters and Sour Orange exhibited the lowest fructose increases (11.59 and 10.16 mg g^−1^ DW, respectively) (Table 2). Sucrose content increased significantly under FZS across all rootstocks, but the magnitude of change varied. UFR5 had the highest sucrose accumulation (16.47 mg g^−1^ DW), followed by UFR07TC (15.87 mg g^−1^ DW). In comparison, Bitters and Sour Orange accumulated comparatively lower sucrose (9.21 and 9.01 mg g^−1^ DW, respectively). Total soluble sugars increased markedly under FZS across all rootstocks. UFR5 displayed the highest accumulation (188.42 mg g^−1^ DW), followed by UFR07TC (171.59 mg g^−1^ DW) and UFR09TC (151.29 mg g^−1^ DW). By contrast, Bitters, Blue-1, and Sour Orange exhibited the lowest increases (112.64, 113.67, and 109.34 mg g^−1^ DW, respectively).

Interestingly, starch reserves declined under FZS in most rootstocks, indicating remobilization into soluble sugars. UFR5 (247.5 to 198.25 mg g^−1^ DW), UFR07TC (243.5 to 194.5 mg g^−1^ DW), and UFR09TC (231.5 to 163.25 mg g^−1^ DW) exhibited significant reductions, although they maintained higher starch than sensitive genotypes (Table 2). The lowest post-freeze starch levels were recorded in Sour Orange (116.25 mg g^−1^ DW) and Bitters (123.75 mg g^−1^ DW). Overall, these results indicate that freezing stress triggers extensive carbohydrate reprogramming in citrus rootstocks.

### 2.9. Correlation Network and Structural Equation Modeling Reveal Integrative Regulation of Freezing Tolerance by Rootstock–Scion Interactions

**Figure 6:**
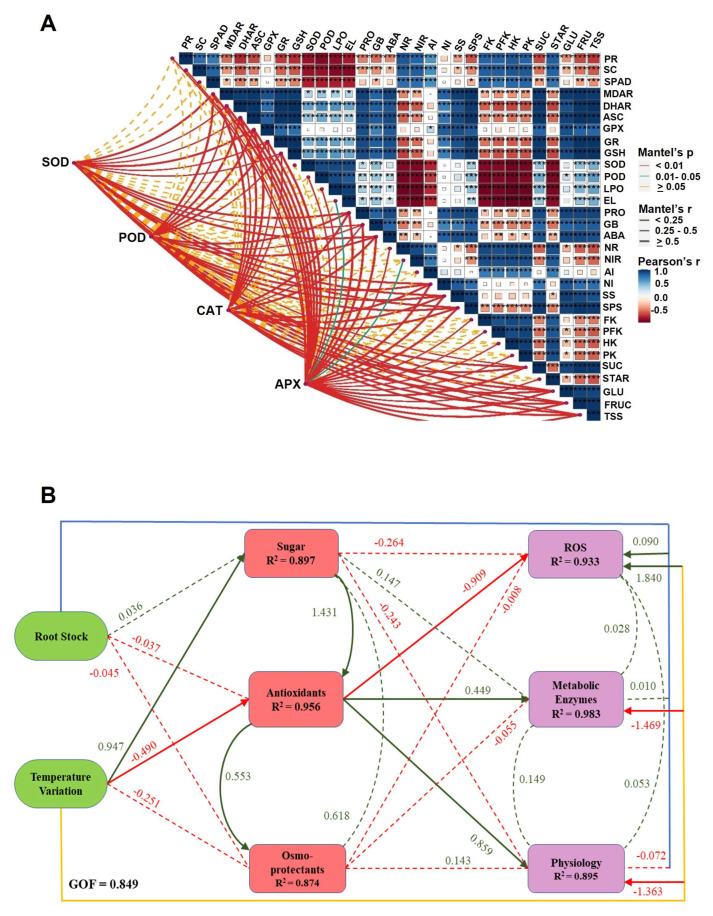
Correlation network and partial least squares structural equational model (PLS-SEM) showing integrative regulation of freezing tolerance in citrus rootstocks. (**A**) Correlation network among physiological, biochemical, and metabolic traits in UF950 scions grafted onto eight rootstocks under control (20 °C) and freezing (−6 °C) conditions. Positive correlations are shown in red and negative correlations in Blue-1, with edge thickness proportional to correlation strength. Antioxidant enzymes (SOD, CAT, APX, GPX, GSH) and osmolytes (proline, GB) were strongly and positively associated with sucrose metabolism enzymes (AI, NI, SuSy, SPS) and glycolytic enzymes (HK, FK, PFK, PK), while negatively correlated with oxidative stress markers (H_2_O_2_, O_2_^•–^, MDA, EL). The asterisk (*) showed the significance level as *p* < 0.05 (* significant), *p* < 0.01 (** more significant), and *p* < 0.001 (*** highly significant). (**B**) Partial least squares structural equational model (PLS-SEM) evaluating the direct and indirect effects of diverse types of rootstocks and temperature levels on physiological and biochemical traits, as well as on the metabolic enzyme activity of leaves of scion UF950. Each rectangular box symbolizes a latent variable. Whereas path lines (green lines for positive and red lines for negative) represent the path coefficient of the latent variable. GOF indicates the goodness of fit statistic of the model, and R^2^ indicates the degree elucidated by their independent latent variables.

To dissect the relationships among physiological, biochemical, and metabolic traits underlying freezing stress tolerance in citrus, we performed a correlation network analysis and partial least squares path modeling (PLS-PM) (Figure 6). The correlation matrix demonstrated a strong positive association between antioxidant enzymes (SOD, POD, CAT, APX) and osmoprotectants (proline, GB), which were in turn negatively correlated with oxidative stress markers, including H_2_O_2_, O_2_^•−^, LPO, and electrolyte leakage (EL). Antioxidant-related variables clustered closely with sucrose metabolism enzymes (SPS, SuSy, NI, AI) and glycolytic enzymes (FK, PFK, PK), suggesting that enhanced carbohydrate metabolism supports the antioxidant defense machinery under freezing stress (Figure 6A). Significant positive correlations were also detected between sugar metabolites (sucrose, glucose, fructose, starch, TSS) and both antioxidant capacity and osmolyte accumulation, reinforcing their dual role as energy substrates and cryoprotectants. Conversely, ROS accumulation and lipid peroxidation showed strong inverse correlations with chlorophyll content (SPAD), photosynthetic performance, and metabolic enzymes, indicating that uncontrolled ROS production is a central determinant of freeze-induced damage (Figure 6A).

Structural equation modeling provided further mechanistic insight into the hierarchical regulation of freezing tolerance (Figure 6B). Rootstock genotype and temperature variation exerted strong direct effects on antioxidant activity (R^2^ = 0.956) and osmoprotectants accumulation (R^2^ = 0.874), which subsequently mediated improvements in physiological stability (R^2^ = 0.895). Antioxidants were the most influential determinant, exerting a significant adverse effect on ROS accumulation (R^2^ = 0.933), while positively influencing metabolic enzyme activity (R^2^ = 0.983). Osmo-protectants, particularly proline and GB, further contributed to sustaining physiological performance under freezing conditions. The overall goodness of fit (GOF = 0.849) validated the robustness of the model, confirming that coordinated regulation of antioxidants, osmoprotectants, and carbohydrate metabolism explains most of the variation in freezing stress tolerance across rootstock–scion combinations (Figure 6B).

### 2.10. Principal Component Analysis

**Figure 7:**
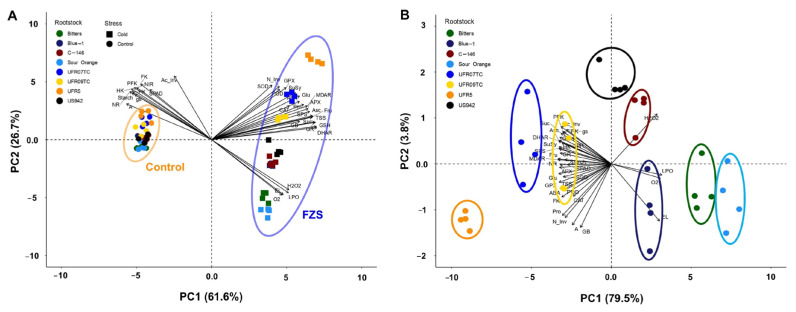
Principal component (PC) analysis of physiological, biochemical, and metabolic traits in UF950 scions grafted onto eight rootstocks. (**A**) PCA showing the traits separation based on the temperature level [control (20 °C) and freezing (−6 °C)]. (**B**) PCA showing the traits separation based on rootstock type. The variables are as follows: net CO_2_ assimilation rate (*A*), stomatal conductance to water vapor (*g*_s_), greenness intensity (SPAD), superoxide ion (O_2_^•−^), hydrogen peroxide (H_2_O_2_), lipid peroxidation (LPO), electrolyte leakage (EL), superoxide dismutase (SOD), peroxidase (POX), catalase (CAT), ascorbate peroxidase (APX), monodehydroascorbate reductase (MDAR), dehydroascorbate reductase (DHAR), glutathione peroxidase (GPX), and glutathione reductase (GR); glutathione (GSH), ascorbate (Asc), proline (Pro), glycine betaine (GB), abscisic acid (ABA), nitrate reductase (NR), nitrite reductase (NIR), acid invertase (AI), neutral invertase (NI), sucrose synthase (SuSy), sucrose-phosphate synthase (SPS), fructokinase (FK), phosphofructokinase (PFK), hexokinase (HK), pyruvatekinase (PK)], sucrose (Suc), glucose (Glu), fructose (Fru), starch, and total soluble sugars (TSS)] on leaves of eight citrus rootstock submitted to freezing stress (−6 °C). The percentages of total variance represented by PC1 and PC2 are shown in parentheses.

To better visualize and understand the differences among citrus rootstocks and their response to freezing stress, a principal component (PC) analysis was performed (Figure 7). The first two PC1 (61.6%) and PC2 (26.7%), explained 88.3% of the total variance and primarily separated citrus rootstocks under control and freeze conditions (Figure 7A). A compacted cluster gathered all citrus rootstocks and correlated positively with *A*, *g*_s_, and SPAD index parameters, as well as starch level and NR, NIR, PFK, HK, FK, and PK activities. Meanwhile, negative correlations between control citrus rootstocks and variables associated with oxidative stress (O_2_^•−^, H_2_O_2_, EL, and LPO) were determined (Figure 7A). On the other hand, citrus rootstocks submitted to freezing stress showed a differential behavior regarding parameters and variables analyzed in this study. To better visualize and understand the differences among citrus rootstocks under freezing stress, another PCA was performed, which shows that PC1 (79.5%) and PC2 (3.8%) explained 83.3% of the total variance (Figure 7B). UFR5, UFR07TC, and UFR09TC rootstocks correlated positively with several components associated with the antioxidant system, like SOD, POD, CAT, APX, GPX and GSH activities, and negatively correlated with EL, O_2_^•−^, and LPO, mainly. Enzymatic activities related to carbon (SuSy, SPS, FK, and PFK) and nitrogen (NR and NIR) correlated positively with UFR5, UFR07TC, and UFR09TC rootstocks. For those rootstocks, photosynthetic parameters (*A* and *g*_s_) and Fru, Glu, and Suc levels were also highly correlated. In contrast, the cellular damage determined by EL, LPO, and oxidative stress was positively correlated with Sour Orange, Bitters, and Blue-1 rootstocks. US942 and C-146 rootstocks did not correlate with some parameters and/or variables and kept an intermediate position between rootstocks with high antioxidative capacity (UFR5, UFR07TC, and UFR09TC) and high cellular damage (Sour Orange, Bitters, and Blue-1) (Figure 7B).

## 3. Discussion

In citrus cultivation, trees are typically established as scion–rootstock combinations, making rootstock selection a critical strategy for enhancing scion resilience to environmental stresses and minimizing yield losses [37]. Moreover, rootstock growth and stress resistance are strongly influenced by scion traits [38], highlighting the importance of scion–rootstock interactions in determining plant performance under adverse conditions. Among abiotic stresses, unexpected freezing events represent a severe constraint, especially in subtropical and tropical regions where citrus is extensively grown. Exposure to freezing temperatures disrupts bioenergetic reactions in plant cells, leading to the overproduction of reactive oxygen species (ROS), which in turn cause membrane lipid peroxidation, structural damage, and alterations in physiological and biochemical processes [12,39]. In stress physiology studies, ROS accumulation is widely recognized as a key indicator of plant sensitivity or tolerance under extreme freezing conditions. An efficient antioxidant defense system is essential for scavenging ROS and mitigating freezing-induced cellular injury [40,41].

The present study demonstrates that scion–rootstock combinations significantly influence freezing tolerance in citrus through their effect on physiological and biochemical responses. Combinations exhibiting higher sensitivity to freezing stress (FZS) showed excessive ROS accumulation, greater membrane damage, diminished antioxidant activity, reduced photosynthetic capacity, and lower carbohydrate reserves. In contrast, cold-tolerant combinations maintained robust antioxidant systems, accumulated higher proline levels, and preserved both the structural integrity and functional performance of photosynthesis, as well as carbon and nitrogen metabolism. Notably, the UF950 scion grafted onto UFR5, UFR07TC, and UFR09TC rootstocks displayed favorable biochemical attributes associated with enhanced freezing tolerance.

For fruit crops, as the fundamental driving force for plant development, fruit yield and quality are influenced by the photosynthetic rate. In previous studies, photosynthetic activity has been widely used as a critical physiological characteristic [42]. ROS can trigger stress signaling pathways that divert energy and resources away from photosynthesis and toward stress repair, resulting in a reduction in photosynthetic activity [43,44]. A reduced supply of water and light under freezing stress causes the downregulation of stomatal opening and closing, leading to a decline in CO_2_ fixation and photosynthetic activity [45]. In citrus trees, photosynthetic characteristics are significantly affected by rootstocks. Studies performed on plants under cold stress have shown that the photoinhibition and stomatal closure processes are induced, reducing light energy dissipation through the photochemical pathway, as well as CO_2_ uptake and assimilation [9,46]. Xie et al. [47] in their study on different citrus scion phenotypes observed that the rate of photosynthesis decreases under stress conditions, and they linked this with blockage between the scion and rootstock communication. Their findings were similar to our results, showing that photosynthetic activity is reduced in plants (Table 1). The decrease in photosynthetic activity (*A* and *g*_s_) under freezing stress can be attributed to stomatal limitations, as the CO_2_ supply from the atmosphere to carboxylation sites in the chloroplast is affected by low *g*_s_ values, limiting the CO_2_ assimilation. In citrus plants submitted to cold stress (4–7 °C), reduction in *A* values were attributed to alterations in the balance between *g_s_* and CO_2_ internal concentration (Ci), suggesting that the stomatal conductivity is a crucial limitation for the CO_2_ assimilation closely linked to photosynthetic enzymes such as Rubisco [46].

In citrus, previous studies have demonstrated significant changes in the cell membrane structure and lipid composition of plants subjected to low temperatures, causing perturbations that lead to ion losses, cellular plasmolysis, and ultimately, functional collapse in the whole plant due to lipid peroxidation (LPO) and ROS generation [48]. Our study depicted that FZS triggered excessive production of ROS (H_2_O_2_ and O_2_^•−^), resulting in electrolyte leakage (EL) and lipid peroxidation (LPO) across all grafted rootstock combinations (Figure 1). However, the UF950/UFR5 (scion–rootstock) combination displayed lower generation of ROS, EL, and LPO compared to other scion–rootstock combinations, suggesting that this scion–rootstock combination exhibits lower structural damage, indicating tolerance. Our results align with those of Tajvar et al. [49], who observed that cold stress increases the rate of LPO in all scion–rootstock combinations, with trifoliate orange exhibiting lower LPO levels compared to other combinations.

Plant tolerance to oxidative damage may be associated with their ability to eliminate ROS. The antioxidant mechanisms include detoxification enzymes such as SOD, CAT, and APX [50]. Under cold stress, plants initiate the activation of antioxidant enzymes (SOD, POD, CAT, APX, GPX, and GSH) to mitigate the adverse effects of ROS in plants [51,52]. These enzymes function as scavengers of reactive oxygen species (ROS), helping to repair ROS-induced membrane damage, reduce electrolyte leakage, and mitigate oxidative damage [44,53]. The SOD activity serves as the first line of defense against ROS by converting superoxide radicals (O_2_^−^) into hydrogen peroxide (H_2_O_2_) and water, while APX, CAT, GPX, and POD are involved in the detoxification of H_2_O_2_ [54]. On the other hand, MDHAR facilitates the regeneration of ascorbic acid (AA) from the unstable intermediate monodehydroascorbate (MDHA), utilizing NADPH as a reducing agent to sustain the cellular AA levels [55]. Meanwhile, DHAR catalyzes the conversion of dehydroascorbate (DHA) back to ascorbic acid (AA), utilizing reduced glutathione (GSH) as the electron donor [56]. Oustric et al. [57] performed an experiment on citrange rootstock (*Citrus sinensis* Osb. × *Poncirus trifoliata* L. Raf.) under freezing stress where they observed an increased SOD, POD, CAT and APX activity in plants under freezing stress, and is similar to the results we obtained in our UFR950 plants grafted onto eight different rootstocks with UFR5 showing better results than other scion–rootstock combinations (Figure 2). This was because the UFR5 rootstock exhibited an efficient antioxidant signaling pathway that activated antioxidant responses in the leaves of the UF950 scion, protecting against freezing stress-induced damage [58].

To mitigate cellular damage caused by environmental stress, plants accumulate compatible solutes, or osmolytes, which stabilize and protect cellular structures. Key osmolytes involved in osmoregulation include proline, glycine betaine (GB), polyamines, and various sugars [59]. The accumulation of proline and GB under stress conditions is widely recognized as an indicator of tolerance, underscoring their critical role in the plant stress response [60]. Proline, with its antioxidant properties, helps scavenge ROS generated in response to freezing stress, thereby enhancing the defense system in plants [61]. In our study, osmolytes such as glucose, sucrose, starch, proline, GB, fructose, and ascorbic acid were present at significantly higher concentrations under freezing stress (Figure 3 and Table 2). These results are consistent with the findings of Saini [62], who demonstrated that frost-tolerant apple (*Malus domestica* Borkh.) cultivars Fuji and Gala grafted onto various rootstocks maintained stable levels of soluble sugars such as sucrose, glucose, and sorbitol during frost-sensitive stages, thereby improving osmoprotection and energy balance. Similarly, Prinsi et al. [63] reported increased total soluble sugar and amino acid levels in the roots and leaves of *Vitis vinifera* Cabernet Sauvignon and in Cab grafted onto the rootstocks 101.14 Millardet et de Grasset (*V. riparia* × *V. rupestris*) or M4 [a hybrid genotype from (*V. vinifera* × *V. berlandieri*) × V. berlandieri Resseguier n.1], when subjected to drought stress.

Cold stress disrupts nitrogen metabolism by inhibiting key enzymes such as nitrate reductase (NR) and nitrite reductase (NIR) through protein denaturation and stress-induced metabolite accumulation [64]. In our study, NR and NIR activities decreased significantly under freezing stress (FZS), but the UF950/UFR5 combination exhibited smaller reductions than other scion–rootstock pairs, indicating a positive interaction that supports nitrogen assimilation (Figure 4). This stability reflects improved protein protection, oxidative stress mitigation, and metabolic homeostasis. Similar patterns were observed in grafted tomato plants, where chilling-tolerant rootstocks or scions maintained higher NR, glutamine synthetase (GS), and glutamate synthase (GOGAT) activities, demonstrating the role of genotype in preserving nitrogen metabolism under low temperatures [65]. The superior performance of UF950/UFR5 is consistent with the known physiological traits of UFR5, including efficient water and nutrient transport, strong antioxidant defenses, and effective stress signaling [66].

Carbohydrate metabolism is a major metabolic pathway in plants. Changes in growth and developmental processes directly influence the transformation of photosynthetic products and protein synthesis [67]. In our study, the activities of acid invertase (AI) were significantly higher than those of neutral invertase (NI) (Figure 5 and Supplementary Table S2). Roitsch and González [68] suggested that AI, localized in the vacuole, may regulate osmosis and cell enlargement, whereas the specific functions of NI remain unclear. This indicates that AI plays a crucial role in sucrose metabolism in Citrus reticulata under freezing stress. Our results show a decline in AI activity alongside an increase in NI activity in citrus plants grafted onto various scion–rootstock combinations. This, together with the concomitant sucrose accumulation observed previously, supports the suggestion that AI activity is negatively correlated with sucrose levels in the leaves [69]. These findings align with those of Xing et al. [70], who studied cucumber (*Cucumis sativus* L. Jinyou No.3) as the scion grafted onto pumpkin rootstock under salt stress, reporting similar alterations in invertase activities under stress conditions.

The highest activities of sucrose synthase (SS) and sucrose phosphate synthase (SPS) were observed in the UF950/UFR5 grafted combination under freezing stress (Figure 5 and Supplementary Table S2). Increased SS and SPS activities under such stress help maintain energy production and prevent excessive sugar accumulation, thereby enhancing cold tolerance. The UFR5 rootstock positively influences these enzyme activities in the UF950 scion, enabling better regulation of sugar metabolism and improved stress resilience [71]. In contrast, the sour orange rootstock showed reduced SS and SPS activities under freezing stress in the UF950 scion, correlating with greater cold sensitivity. These results concur with those of Liang et al. [72], who reported increased SS and SPS activities in bitter gourd (*Momordica charantia* L. F-1437) grafted onto different rootstocks of pumpkin (*Cucurbita moschata*) and luffa (*Luffa cylindrica*) under heat stress. The enhanced enzyme activities contributed to improved carbon metabolism and stress tolerance in grafted bitter gourd seedlings.

The activities of hexokinase (HK), phosphofructokinase (PFK), fructokinase (FK), and pyruvate kinase (PK) decreased in all tested rootstocks grafted with the UF950 scion under freezing stress (Figure 5 and Supplementary Table S2). These enzymes play essential roles in glycolysis and pyruvate production [73]. The lowest enzyme activities were observed in the sour orange grafted rootstock. However, the UF950/UFR5 grafted combination exhibited the least decline in these enzyme activities under freezing stress compared to control conditions, demonstrating superior metabolic stability. The upregulation of these responses in the UF950 scion is attributed to the UFR5 rootstocks consistent performance under cold hardiness, efficient water and nutrient uptake, and stronger union with the grafted scion, likely due to specific gene expression patterns distinguishing it from other rootstock varieties [74,75]. Shahid et al. [76] reported similar findings in their study, where the activities of HK, FK, PFK, and PK were increased in three citrus rootstock species *Poncirus trifoliata* (PT), *Citrus reshni* (CH), and *Citrus limonia* Osbeck (CL) under chromium stress. This can be attributed to the role of hexokinase (HK) as a sugar sensor that regulates the expression of a wide range of genes, as reported by Granot et al. [77]. Similarly, fructokinase (FK) also functions as a sugar sensor in plant tissues; however, Gupta and Kaur [78] noted that FK operates via pathways independent of those involving HK.

**Figure 8:**
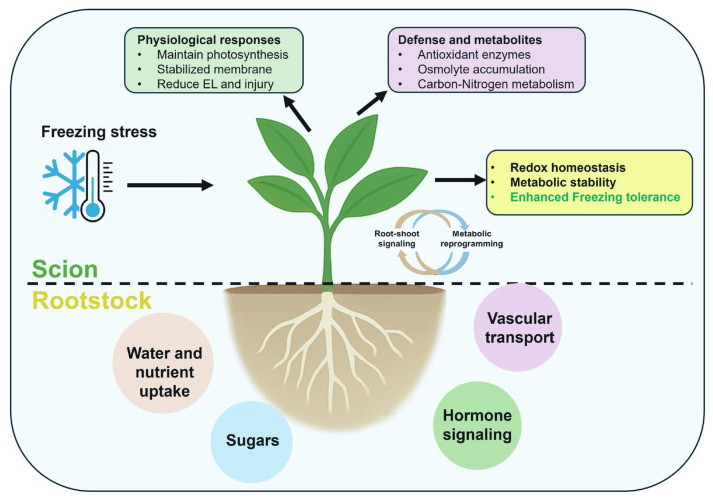
Mechanistic diagram of how tolerant rootstocks enhance scion freezing resilience by integrating redox homeostasis, osmolyte accumulation, and carbon–nitrogen metabolic reprogramming.

The network further revealed a close linkage between carbohydrate metabolism and oxidative stress regulation, with sucrose metabolism enzymes (AI, NI, SuSy, SPS) and glycolytic enzymes (HK, FK, PFK, PK) clustering alongside antioxidants and osmolytes (Figure 6). This suggests that soluble carbohydrate turnover not only provides substrates for energy metabolism but also supports antioxidant defense and osmotic adjustment, consistent with studies in grapevine and apple demonstrating that sugar accumulation acts as both a metabolic fuel and cryoprotectant [79,80]. SEM analysis reinforced these interactions, showing that antioxidant activity exerted the most decisive influence on reducing ROS accumulation (R^2^ = 0.933) while positively driving metabolic enzyme activity (R^2^ = 0.983). Osmolyte accumulation further stabilized physiological performance (R^2^ = 0.895), indicating that the interplay between redox regulation and osmotic adjustment is essential for sustaining photosynthetic function and membrane stability under freezing stress. Notably, rootstock genotype emerged as a key driver of these pathways, supporting the hypothesis that root-to-shoot signaling, and metabolic reprogramming underlie scion tolerance [20,81]. The conceptual model illustrates how tolerant rootstocks enhance scion freezing resilience by integrating redox homeostasis, osmolyte accumulation, and carbon–nitrogen metabolic reprogramming. Through coordinated root-to-shoot signaling, these pathways converge to maintain photosynthetic efficiency, stabilize cellular membranes, and minimize oxidative injury, thereby enabling sustained performance under freezing stress (Figure 8). The outcomes of this study extend beyond fundamental physiology by offering practical strategies for citrus improvement in frost-prone regions. The identified scion–rootstock combinations that optimize antioxidant defense and carbon–nitrogen metabolic balance can serve as key selection criteria for nurseries and growers. Integrating these traits into breeding and extension programs will accelerate the adoption of resilient grafts, minimize frost-related yield losses, and promote sustainable production. Such targeted use of scion–rootstock interaction provides a cost-effective and environmentally friendly alternative to chemical or energy-intensive frost protection methods.

## 4. Materials and Methods

### 4.1. Plant Material, Growth Conditions, and Freezing Treatment

Two-year-old UF950 (*Citrus reticulata*) plants were bud-grafted on to eight different rootstocks (Bitters, Blue-1, C-146, Sour orange, UFR07TC, UFR09TC, UFR5, and US942) were used as an experimental material and grown in the greenhouse facility of Horticultural Science Department at North Florida Research and Education Center, Quincy, University of Florida, (27°25′32.6604″ N; 80°24′18.7092″ W). The plants were grown in specialized square plastic pots (4 × 13.5 inches) known as citri pots filled with coconut fiber growing media. The plants were kept in a controlled greenhouse, where they were maintained at an average day/night temperature of 22 ± 2 °C, with 80% relative humidity, and a photoperiod of 12 h for three weeks to facilitate optimal growth. Plants were fertigated with fertilizer (10:10:10) NPK with micros at 150 ppm once a week. After six weeks, the plants were shifted into a programmed walk-in-freezing chamber (Conviron, CMP 6050, Winnipeg, MB, Canada) facility at the Horticulture Sciences Department, University of Florida, Gainesville, United States (29°38′23″ N 82°21′35″ W). All plants were placed under normal temperature 20 °C (control), for 24 h to acclimate and then exposed to freezing temperature (−6 °C). The freezing treatment involved a controlled temperature reduction in the cold chamber at a rate of 2 °C per hour, beginning at 20 °C and continuing until a target temperature of −6 °C was achieved. This final temperature was then maintained for 1.5 h to complete the exposure. The relative humidity in the freezing chamber was 95%. The selection of this freezing temperature was based on the previous study done by this lab [12]. After the freezing treatment, leaf samples were collected in liquid nitrogen and shifted to −80 °C for analysis.

### 4.2. Gas Exchange Parameters and SPAD Measurements

Leaf gas exchange parameters were measured using a portable photosynthesis system (LI-6800; LI-COR Inc., Lincoln, NE, USA). Measurements were conducted under two temperature regimes, 20 °C (control) and −6 °C (freezing stress), using fully expanded, healthy leaves from three different canopy positions of each plant as elaborated by Iqbal et al. [82]. In addition, the photosynthesis instrument conditions were as follows: the molar airflow per unit leaf was 395 mmol m^−2^ s^−1^, air CO_2_ concentration was 380 µmol mol^−1^, leaf temperature 25 °C, relative humidity (70–80%), and light intensity (500 µmol m^−2^ s^−1^). Leaf greenness was assessed using a Soil–Plant Analysis Development (SPAD) chlorophyll meter (SPAD-502 Plus, Konica Minolta Inc., Tokyo, Japan). Measurements were recorded from 15 fully expanded mature leaves per plant for each treatment, ensuring uniform sampling from all sides of the plant canopy.

### 4.3. Reactive Oxygen Species, Electrolyte Leakage, and Lipid Peroxidation

Reactive oxygen species and lipid peroxidation analyses were conducted following the methods described by Elstner and Heupel [83]. Briefly, superoxide (O_2_^•−^) content was determined using nitroBlue-1 tetrazolium (NBT) reduction, and absorbance was measured at 540 nm. Hydrogen peroxide (H_2_O_2_) and malondialdehyde (MDA) contents were quantified using commercial assay kits (Elabscience, Houston, TX, USA) following the manufacturer’s instructions. Results were normalized to fresh weight concentration and expressed as nm g^−1^ FW (O_2_^•−^, H_2_O_2_) or U min^−1^ g^−1^ FW (MDA). For electrolyte leakage (EL), leaves (fresh afterwards stored at −80 °C) were cut into pieces and immersed in 10 mL of distilled water and incubated at 25 °C for 2–3 h. The initial EL was then measured using a conductivity meter (Orion VersaStar Pro, Thermo Scientific, Jakarta, Indonesia). Afterward, the samples were kept in a water bath at 98 °C for 45 min to ensure complete membrane disruption, and then the second EL was performed. The EL (%) was determined using the formula [EL (%) = Initial EC/Second EC × 100].

### 4.4. Antioxidant Enzymes and Osmolytes

The activities of antioxidant enzymes such as superoxide dismutase (SOD), peroxidase (POD), catalase (CAT), ascorbate peroxidase (APX), glutathione peroxidase (GPX) and glutathione (GSH), as well as the concentrations of proline (Pro) and glycine betaine (GB), were determined following the protocols described by Iqbal and Shahid et al. [76,84]. Fresh leaf tissues were homogenized in extraction buffer, centrifuged, and the supernatants were used for analysis. Enzyme activities were measured spectrophotometrically and expressed as U mg^−1^ protein, while proline and glycine betaine contents were quantified as mg g^−1^ fresh weight based on standard curves.

### 4.5. Nitrogen Metabolism Enzymes

Nitrate reductase (NR) and nitrite reductase (NiR) activities were determined following the protocols described by Shahid et al. [76]. For NR activity, 1 g of fresh leaf tissue was incubated in a reaction mixture containing phosphate buffer (pH 7.0), KNO_3_, sulphanilamide, and N-(1-naphthyl)-ethylene diamine dihydrochloride, and centrifuged at 14,000× *g* for 5 min at 4 °C. The absorbance of the resulting solution was measured at 542 nm, and NR activity was expressed as µmol NO_2_^−^ g^−1^ FW h^−1^. For NiR activity, 1 g of frozen leaf tissue was extracted in phosphate buffer (pH 5.0) containing NaNO_2_, and the reaction was developed by adding sulphanilamide and N-(1-naphthyl)-ethylene diamine dihydrochloride. After 30 min of color development, absorbance was measured at 540 nm. NiR activity was expressed as µmol NO_2_ g^−1^ FW h^−1^.

### 4.6. Carbon Metabolic Enzymes

The enzymatic activities related to carbohydrate metabolism were assessed following the protocol established by Iqbal et al. [82]. Briefly, 0.5 g of leaf tissue was homogenized in an extraction buffer containing Tris-HCl, MgCl_2_, β-mercaptoethanol, EDTA, EGTA, 2-hydroxyethanethiol, and glycerol, and centrifuged at 17,000× *g* for 15 min at 4 °C. The supernatant was used for enzymatic assays.

Total acid invertase (AI) and neutral invertase (NI) activities were determined using specific buffer systems (pH 4.8 for AI and phosphate-citrate for NI) with sucrose as the substrate. After incubation at 37 °C for 40 min, the reactions were terminated with dinitrosalicylic acid, and the absorbance was measured at 540 nm. Sucrose synthase (SuSy) and sucrose phosphate synthase (SPS) activities were measured according to Rufty Jr., while fructokinase (FK) activity followed the method of Zrenner et al. [85]. Hexokinase (HK), phosphofructokinase (PFK), and pyruvate kinase (PK) activities were determined using commercial assay kits (Sigma-Aldrich, St. Louis, MO, USA) according to the manufacturer’s instructions. All enzyme activities were quantified as µmol min^−1^ mg^−1^ fresh weight (FW), with one unit defined as the amount of enzyme catalyzing the conversion of 1 µmol of substrate per minute per milligram of FW at 37 °C.

### 4.7. Quantification of Sugar and Starch Concentrations

The concentrations of soluble sugars and starch were quantified using a microplate reader (ELx800-ts, BioTek Instruments, Winooski, VT, USA) following the protocols provided with commercial assay kits. For each assay, 10 mg of finely powdered tissue (ground in liquid nitrogen) was extracted according to the manufacturer’s instructions, and ~200 µL of the reaction mixture was transferred into a 96-well microplate. Absorbance was measured at the following wavelengths using the respective kits: glucose—570 nm (Sigma-Aldrich, MAK476), starch–570 nm (Sigma-Aldrich, MAK368), fructose—340 nm (Sigma-Aldrich, FA20), and sucrose—340 nm (Sigma-Aldrich, SCA20). The concentrations of soluble sugars and starch were expressed on a dry weight (DW) basis (mg g^−1^ DW), representing the milligrams of sugar or starch per gram of oven-dried tissue, thereby normalizing for differences in water content.

### 4.8. Experimental Design and Statistical Analysis

A two-by-two factorial was used for this experiment, consisting of eight different rootstocks (Bitters, Blue-1, C-146, Sour orange, UFR07TC, UFR09TC, UFR5, and US942) and two temperature levels (20 °C and −6 °C), arranged in a completely randomized design with four and 10 plants per replication. Before ANOVA, data were assessed for normality using the Shapiro–Wilk test and for homogeneity of variances using Levene’s test. Where assumptions were not met, data were log- or square-root transformed before analysis. The physiological and biochemical data set was subjected to an analysis of variance [ANOVA; by *F* test (*p* ≤ 0.05)] to evaluate the interaction between citrus rootstocks and temperature levels, followed by Tukey’s test (*p* < 0.05) to better understand the differences between the citrus rootstocks for each temperature level. Principal component analysis (PCA) was applied to log2-transformed data to investigate the relationship between physiological and biochemical variables, parameters, and the evaluated factors. Data analysis was performed using RStudio software version 4.0.4 (R Core Team, 2021) with the packages “*factoextra*”, “*agricolae*”, “*ggplot2*”, and “*corrplot*” [86,87,88].

## 5. Conclusions

This study demonstrates that scion–rootstock interaction is critical for enhancing freezing tolerance in *Citrus reticulata* by integrating photosynthetic efficiency, redox balance, osmolyte accumulation, and carbon–nitrogen metabolic regulation. The UF950/UFR5 combination showed superior performance under freezing stress, with elevated antioxidant enzyme activities, increased proline and glycine betaine, sustained sugar metabolism, and minimal cellular damage, preserving chloroplast function and photosynthesis. Robust rootstocks (UFR5, UFR07TC, UFR09TC) orchestrated systemic stress responses through efficient root-to-shoot signaling and metabolic regulation, whereas freeze-sensitive rootstocks (e.g., Sour Orange) failed to maintain homeostasis. These findings underscore the pivotal role of rootstock genotype in scion resilience and identify UF950/UFR5 as a promising combination for breeding freeze-tolerant citrus for sub-tropical and temperate regions facing climate variability.

## Figures and Tables

**Table 1:** Average values of net carbon assimilation rate (*A*; µmol photons m^−2^ s^−1^), stomatal conductance to water vapor (*g*_s_; mol CO_2_ m^−2^ s^−1^), and leaf greenness (SPAD), on leaves of eight citrus rootstock submitted to two temperatures levels [20 °C (control) and −6 °C (freezing stress)]. Average values with an asterisk (*) differ significantly (*p* ≤ 0.05) between temperature levels according to the F test. Meanwhile, rootstock average values for each temperature level followed by different letters are significantly different according to Tukey’s test (*p* ≤ 0.05); *n* = 4.

	*A*		*g*_s_		SPAD	
Rootstock	Control	FZS	Control	FZS	Control	FZS
Bitters	24.5 a*	12.4 cd	54.0 a*	24.5 cd	46.4 a*	31.6 c
Blue 1	23.1 a*	15.9 abc	51.8 a*	30.3 bcd	50.3 a*	35.1 bc
C146	20.9 a*	11.4 de	51.9 a*	36.8 abc	50.3 a*	37.8 abc
Sour Orange	23.0 a*	8.3 e	51.5 a*	20.5 d	47.1 a*	29.4 c
UFR07TC	25.4 a*	13.9 bcd	54.3 a*	40.2 ab	54.4 a*	45.7 ab
UFR09TC	24.6 a*	17.1 ab	53.4 a*	39.8 ab	49.3 a*	40.2 abc
UFR5	23.5 a*	18.5 a	60.3 a*	44.8 a	54.1 a*	48.2 a
US942	23.5 a*	11.4 de	52.9 a*	36.0 abc	45.1 a*	34.4 bc

**Table 2:** Average values of glucose (mg g^−1^ DW), fructose (mg g^−1^ DW), sucrose (mg g^−1^ DW), starch (mg g^−1^ DW), and total soluble sugars (TSS; mg g^−1^ DW) concentrations on leaves of eight citrus rootstock submitted to two temperatures levels [20 °C (control) and −6 °C (freezing stress; FZS)]. Average values with an asterisk (*) differ significantly (*p* ≤ 0.05) between temperature levels according to the F test. Meanwhile, rootstock average values for each temperature level followed by different letters are significantly different according to Tukey’s test (*p* ≤ 0.05). *n* = 4. DW = dry weight.

	Glucose		Fructose		Sucrose		Starch		TSS	
Rootstock	Control	FZS	Control	FZS	Control	FZS	Control	FZS	Control	FZS
Bitters	14.6 bcd	22.5 d*	4.7 ab	11.6 e*	4.3 a	9.2 de*	222 a*	124 de	37.2 ab	113 e*
Blue 1	15.8 bcd	26.2 cd*	3.9 bc	14.4 d*	4.4 a	11.0 e*	225 a*	160 bcd	40.6 a	114 e*
C146	18.6 a	27.4 cd*	5.2 a	15.6 d*	4.8 a	13.0 cd*	225 a*	134 cde	32.8 bc	122 d*
Sour Orange	13.6 d	20.3 d*	4.5 abc	10.2 e*	4.9 a	9.0 e*	212 a *	116 e	38.2 a	109 e*
UFR07TC	17.1 ab	46.4 b*	4.0 bc	19.6 b*	5.3 a	15.9 ab*	244 a*	195 ab	38.0 a	171 b*
UFR09TC	15.0 bcd	42.6 b*	3.4 c	17.8 bc*	5.5 a	13.8 bc*	231 a*	163 abc	39.5 a	151 c*
UFR5	16.7 abc	55.0 a*	5.4 a	23.3 a*	5.0 a	16.5 a*	248 a*	198 a	38.9 a	188 a*
US942	14.4 cd	32.3 c*	5.4 a	16.5 cd*	5.3 a	13.1 cd*	232 a*	167 abc	31.9 c	123 d*

## Data Availability

The data used in this study are presented in the article.

## Supplementary Materials

The following supporting information can be downloaded at https://www.mdpi.com/article/10.3390/plants14193029/s1, Supplementary Table S1. Analysis of variance for the effects of rootstock (RS), stress condition (SC), and the interaction RS × TL on the net CO_2_ assimilation rate (A), stomatal conductance to water vapor (gs), greenness intensity (SPAD), concentrations of superoxide ion (O_2_^−^), hydrogen peroxide (H_2_O_2_), malondialdehyde (LPO), and electrolyte leakage (EL); activities of enzymes involved in the antioxidative metabolism [superoxide dismutase (SOD), peroxidase (POX), catalase (CAT), ascorbate peroxidase (APX), monodehydroascorbate reductase (MDAR), dehydroascorbate reductase (DHAR), glutathione peroxidase (GPX), and glutathione reductase (GR)]; concentration of glutathione (GSH), ascorbate (Asc), proline (Pro), glycine betaine (GB), the activities of enzymes involved in the nitrogen [Nitrate reductase (NR) and Nitrate reductase (NIR)] and carbon [acid invertase, alkaline invertase, sucrose synthase (SuSy), sucrose-phosphate synthase (SPS), Fructokinase (FK), Phosphofructokinase (PPK), Hexokinase (HK), Pyruvatekinase (PK)], and concentrations of carbohydrates [sucrose, glucose, fructose, starch, and total soluble sugars (TSS)] determined in citrus rootstocks. *** ≤ 0.001, ** ≤ 0.01, * ≤ 0.05, and ns = non-significant. Supplementary Table S2. Relative (cold/control) log2-transformed concentration values of AI (acid invertase), NI (neutral invertase), SUSY (sucrose synthase), SPS (sucrose phosphatesynthase), FK (fructokinase), PFK (phosphofructokinase), HK (hexokinase) and PK (pyruvatekinase) on leaves of eight citrus rootstock submitted to two temperatures levels [20 °C (control) and −6 °C (freeze stress; FZS)].
