# Supplementary material for: Scion–Rootstock Interactions Enhance Freezing Stress Resilience in Citrus reticulata Through Integrated Antioxidant Defense and Carbon–Nitrogen Metabolic Adjustments
Source: Plants (Basel). 2025 Sep 30;14(19):3029. doi: 10.3390/plants14193029 (PMC12525908; doi:10.3390/plants14193029)
Supplement: Supplementary file 1 [file plants-14-03029-s001.zip › Supplementary Table S1.pdf]

Supplementary Table S1. Analysis of variance for the effects of rootstock (RS), stress condition (SC), and the interaction RS × TL on the net CO_2 assimilation rate (*A*), stomatal conductance to water vapor (g_s), greenness intensity (SPAD), concentrations of superoxide ion (O_2^-), hydrogen peroxide (H_2O_2), malondialdehyde (LPO), and electrolyte leakage (EL); activities of enzymes involved in the antioxidative metabolism [superoxide dismutase (SOD), peroxidase (POX), catalase (CAT), ascorbate peroxidase (APX), monodehydroascorbate reductase (MDAR), dehydroascorbate reductase (DHAR), glutathione peroxidase (GPX), and glutathione reductase (GR)]; concentration of glutathione (GSH), ascorbate (Asc), proline (Pro), glycine betaine (GB), the activities of enzymes involved in the nitrogen [Nitrate reductase (NR) and Nitrate reductase (NIR)] and carbon [acid invertase, alkaline invertase, sucrose synthase (SuSy), sucrose-phosphate synthase (SPS), Fructokinase (FK), Phosphofructokinase (PPK), Hexokinase (HK), Pyruvatekinase (PK)], and concentrations of carbohydrates [sucrose, glucose, fructose, starch, and total soluble sugars (TSS)] determined in citrus rootstocks. *** = <0.001, ** = <0.01, * = <0.05, and ns = non-significant.

	F values		
Parameter/Variable	RS	SC	RS × SC
A	6.5^{***}	310.8^{***}	4.1^{**}
g_s	5.5^{***}	160.0^{***}	2.5^{*}
SPAD	5.7^{***}	67.9^{***}	0.9^{ns}
$O_2^{\bullet-}$	14.7^{***}	267.6^{***}	9.0^{***}
H_2O_2	25.2^{***}	580.1^{***}	12.9^{***}
LPO	23.5^{***}	620.3^{***}	18.3^{***}
EL	35.9^{***}	718.1^{***}	36.5^{***}
SOD	24.4^{***}	62.9^{***}	3.9^{**}
POD	33.5^{***}	130.6^{***}	7.1^{***}
CAT	5.7^{***}	73.5^{***}	1.1^{ns}
APX	32.1^{***}	873.1^{***}	15.1^{***}
MDAR	81.8^{***}	2002.7^{***}	45.4^{***}
DHAR	93.0^{***}	10959.7^{***}	61.8^{***}
Asc	59.2^{***}	3210.7^{***}	56.7^{***}
GPX	46.1^{***}	253.0^{***}	12.8^{***}
GR	26.6^{***}	2193.5^{***}	23.4^{***}
GSH	47.5^{***}	5322.5^{***}	39.3^{***}
Pro	27.1^{***}	505.5^{***}	14.0^{***}
GB	2.1^{*}	107.2^{***}	1.7^{ns}
NR	25.5^{***}	565.5^{***}	2.0^{ns}
NIR	20.6^{***}	404.5^{***}	10.5^{***}
Ac. Inv.	59.8^{***}	116.9^{***}	2.8^{*}
N. Inv.	50.0^{***}	191.8^{***}	16.2^{***}
SuSy	48.7^{***}	518.7^{***}	24.5^{***}
SPS	32.7^{***}	1171.6^{***}	22.1^{***}
FK	19.6^{***}	338.6^{***}	9.2^{***}
PPK	8.2^{***}	287.8^{***}	6.1^{***}
HK	19.9^{***}	765.8^{***}	22.4^{***}

PyK	21.9***	525.6***	11.9***
Sucrose	27.6***	1390.3***	18.7***
Glucose	56.1***	864.9***	46.1***
Fructose	70.0***	3870.7***	63.6***
Starch	13.4***	324.6***	3.0**
TSS	322.5***	26188.8***	288.1***
