# Supplementary material for: Scion–Rootstock Interactions Enhance Freezing Stress Resilience in Citrus reticulata Through Integrated Antioxidant Defense and Carbon–Nitrogen Metabolic Adjustments
Source: Plants (Basel). 2025 Sep 30;14(19):3029. doi: 10.3390/plants14193029 (PMC12525908; doi:10.3390/plants14193029)
Supplement: Supplementary file 1 [file plants-14-03029-s001.zip › Supplementary Table S2.pdf]

Supplementary Table S2. Relative (cold/control) log2-transformed concentration values of AI (acid invertase), NI (neutral invertase), SUSY (sucrose synthase), SPS (sucrose phosphatesynthase), FK (fructokinase), PFK (phosphofructokinase), HK (hexokinase) and PK (pyruvatekinase) on leaves of eight citrus rootstock submitted to two temperatures levels [20 ºC (control) and –6 ºC (freezing stress; FZS)].

Stress	Cultivar	AI	NI	SUSY	SPS	FK	PFK	HK	PK
Cold/Control	Bitters	-1.14066	0.245112	0.449582	0.783062	-0.74528	-0.88301	-1.21929	-1.0375
Cold/Control	Blue-1	-0.65966	0.236479	0.622907	1.025665	-0.69161	-0.7186	-0.95209	-0.8661
Cold/Control	C-146	-0.63286	0.34853	0.78774	1.174687	-0.62105	-0.60348	-0.8603	-0.55516
Cold/Control	Sour Orange	-1.58496	0.164196	-0.10081	0.912761	-0.87033	-0.9615	-1.63318	-1.16568
Cold/Control	UFR07TC	-0.21175	0.54264	0.891407	1.540974	-0.18862	-0.28927	-0.25412	-0.30003
Cold/Control	UFR09TC	-0.23522	0.603124	0.774371	1.375433	-0.34062	-0.50642	-0.61534	-0.50244
Cold/Control	UFR5	-0.12691	0.751148	1.027068	1.605257	-0.11185	-0.1581	-0.17655	-0.17794
Cold/Control	US942	-0.42742	0.063425	0.848548	1.26942	-0.63883	-0.4426	-0.73255	-0.56607
